# Cost-effectiveness and resource allocation (CERA) 18 years of evolution: maturity of adulthood and promise beyond tomorrow

**DOI:** 10.1186/s12962-020-00210-2

**Published:** 2020-04-02

**Authors:** Mihajlo Jakovljevic, Klazien Matter-Walstra, Takuma Sugahara, Tarang Sharma, Vladimir Reshetnikov, Joav Merrick, Tetsuji Yamada, Sitaporn Youngkong, Joan Rovira

**Affiliations:** 1 Department of Global Health Economics and Policy, University of Kragujevac, Kragujevac, Serbia; 2 Institute of Comparative Economic Studies, Faculty of Economics, Hosei University Tokyo, Tokyo, Japan; 3 Department HTA Swiss Federal Office of Public Health, Bern, Switzerland; 4 Evidence to Policy®, Copenhagen, Denmark; 5 N.A. Semashko Department of Public Health and Healthcare, I.M. Sechenov the First Moscow State Medical University (Sechenov University), Moscow, Russia; 6 Hadassah Hebrew University Medical Center, Division of Pediatrics, Mt Scopus Campus, Jerusalem, Israel; 7 Department of Economics, Rutgers University, New Jersey, USA; 8 Department of Pharmacy, Faculty of Pharmacy, Mahidol University, Bangkok, Thailand; 9 Department of Economic Theory, University of Barcelona, Barcelona, Spain

**Keywords:** Health economics, Health financing, Political economy, Outcomes research, Econometrics, Bibliography, Low- and middle-income countries, Emerging markets, EM7, BRICS, Economic evaluation, Cost-effectiveness analysis, I11, I15, I18

## Abstract

Since its inception in 2003, *Cost Effectiveness and Resource Allocation* journal has come a long way over the past 18 years. Possibly much longer than many of its contemporaries in the blossoming science of health economics might have anticipated. Today, entering 2020 it celebrates the Age of Maturity. We believe that in the third decade of XXI century the interdisciplinary science of health economics, will rejuvenate and come back to us younger than ever from its early historical roots almost a century ago. The spreading of economic globalization in several distinctive ways, either led by multinational business corporations or newly emerged Asian leadership, or both, is likely to make challenges for contemporary health systems far more serious. The fourth industrial revolution (cyber physical systems and artificial intelligence technology) and accelerated innovation in the field of E-Health and digital health, will probably change the workflow in medical and health care, and inevitably transform the labour market in the upcoming decades. So, let us be up to the task. Let us provide academic centres, industry-sponsored pharmaceutical and medical device innovation hubs, and governing authorities alike, with a powerful forum for debate on cost-effective resource allocation in the years to come.

Since its inception in 2003, *Cost Effectiveness and Resource Allocation* (CERA) journal has evolved and grown substantially over the past 18 years. Possibly much longer than many of its contemporaries in the blossoming science of health economics might have anticipated. Today, within the third decade of XXI century it celebrates the Age of Maturity. The published articles since 1st January 2003 were downloaded globally over 189,720 times worldwide [1]. Furthermore if we stratify this large body of contributions, across the countries of origin of manuscripts we come to the reality that the top ten major manuscript contributors were: USA–845; UK–537; Australia–154; Germany–146 Spain–141; Canada–131; South Africa–117; Netherlands–91; Italy–88 and India–73. This refers to entire count of all submissions regardless of their final destiny throughout their publishing pathway being either accepted, rejected or withdrawn. Given the fact, that this is an absolute cross section throughout the entire life span of the Journal, it may be misleading to some extent. Yet in recent years we observe the underlying trend that mainstream of submissions come from the Emerging nations. The South Asian region, Islamic Republic of Iran, Thailand, Malaysia and Brazil are among a few of the prominent contributors and an accelerated workflow originating from Sudanese Africa, with Malawi academic centres being a typical example. This is an encouraging trend, where we can see that the journal provides a mechanism for emerging economies to showcase their experiences of cost-effectiveness studies and resource allocation globally.

When we come up to the most typical areas of exploration of the majority of our manuscripts, even greater surprises are yet to come. Among disease areas some of the leading ones are neoplasms, infectious and parasitic diseases and cardiovascular diseases. According to treatment and technology classifications, key dominant areas of interest were pharmaceuticals to be followed by diagnostic technologies and medicinal devices. Most commonly observed types of interventions in econometric studies were public health, followed by preventive and hospital inpatient medical care. When we take into account the methodological approaches most commonly identified in published articles, we discover that economic evaluations are by far the most common ones, while costing analysis and methodological reviews themselves are appealing to our target audience as well. Last but not least, we may notice that, from a health policy perspective, the most frequent research endeavours are centred around health insurance & financing, to be followed after a large margin by access & equity analysis, cost-effectiveness analysis and price regulation issues. CERA may be proud with its successful build-up of an exceptionally large and diverse reviewer base currently counting at 3059 of experts, whose distribution across the disciplines of health economics and geographic jurisdictions is truly heterogenous.

Observing the current momentum developing over a long course of years, we may say that particular a recent highlight of CERA previous leaderships was its placement at the 35th out of total of 77 best impact journals in the Journal Citation Report ranking for the disciplines of Health Policy & Services back in 2016 [2]. The journal is proud with its current impact of 1.676 situated in the second Quartile (Q2) as per the Journal Citation Report and quite appealing dynamics of its scientometrics indicators in the broader Health Economics and Outcomes Research (HEOR) area [3]. Furthermore, there is a convenient bibliographic cross section of highest impact health economics journals, last updated on 17 July 2018 and compiled by Philip Clarke on behalf of the Mt Hood Diabetes Challenge Network and well-established Editors of Pharmacoeconomics and Applied Health Economics journals published by Adis^®^ and based in New Zealand (Chris Carswell and Tim Wrightson). In this comparative analysis of a total of 31 leading journals, CERA is placed per some of its efficiency criteria as the fourth listed one [4]. These and other successes of the Journal are largely attributable to its pool of prestigious academic editors, but also an excellent technical support to the open access publishing model provided by BMC’s offices [5].

Back in 2009, in the seventh consecutive year of CERA’s academic growth, an excellent editorial article was published depicting a vision that a group of prominent CERA editors had at that time [6]. A decade ago, the journal was approximately 3.5 times smaller than today, but that had clearly accelerated its growth pathway which ultimately culminated with CERA obtaining its first formal impact factor 7 years later. Editors among whom several were affiliated to large multinational health organizations at the time, properly created a vision of a niche journal. They decided CERA’s geographic focus should be to recruit quality and innovative research and broaden its target audience worldwide, to expand its outlook. They shifted away from richest OECD nations who were driving the Journal development in its early years since establishment. The anticipated epicentre of further CERA development should have been centred around diverse and heterogeneous Low-and-Middle-Income countries (LMICs) and their needs for reliable health economic evidence. Why did they make such a choice? At that time the global recession triggered by bankruptcy of Brother Lehman had just began. Academic sources citing early and shy occurrence of world multipolarity and rise of emerging BRICS (Brazil, Russia, India, China, South Africa) and Emerging Seven nations (EM7- China, India, Russia, Brazil, Mexico, Indonesia, Turkey) markets were still rather rare [7]. Inevitably this gradual and slow change reflected on research and development investment in pharmaceutical and medicinal device industry. The flow of innovation hubs and cutting-edge technology patents consecutively shifted away from traditional industrialised Global North towards Global South and primarily Asia [8]. This has had a profound effect on the stream of research in health economics and associated sciences. In bibliographic terms it is enough to follow the line from the massive bibliographic analysis of global HEOR research output published by Adam Wagstaff on behalf of the World Bank in 2011 [9] and the one published by Mihajlo Jakovljevic and Ogura Seiritsu in 2016 [10]. Underlying trends reveal that South Asian nations and China are rapidly becoming the fastest growing global publishing regions in biomedicine and interdisciplinary health sciences alike. Thus, it appears that Rob Baltussen et al. truly had an inspirational vision for CERA in 2009 since their anticipated shift in Journal attention has placed it at the forefront of global development in its associated sciences [11]. Now many years later, their wisdom continues to pay off. The historical cradle of health economics was in US academia [12] and, at a much latter stage, in Western Europe and Japan. Institutional capacities and political administration of this knowledge in formal decision making on resource allocation, remain by far the most advanced in Western nations [13]. That said, the Health Technology Assessment (HTA) network of public agencies (The International Network of Agencies for Health Technology Assessment (INAHTA)) has already grown substantially in the Eastern Europe and Asian Western Pacific countries [14]. Today it appears that the core of the future development in HEOR sciences is gradually moving towards the emerging markets with BRICS [15] and Next Eleven being the most prominent representatives [16]. There is a rather strong degree of saturation among Western academic networks with a traditional HEOR publishing platforms ranging from academic journals to blogs, public repositories and industry sector influenced grey literature sources [17]. This hierarchy of evidence is well established [18]. It has survived a test of time in free-market economies ranging from the early theoretical milestone papers by Milton Friedman back in 1930s [19] and Selma Mushkin back in 1958 [20] (pioneering article: “*Toward a definition of health economics*”). This hierarchy has survived and strengthened itself within a capitalism-driven socioeconomic system throughout three major recessions, two world wars, Cold War era and ultimately an accelerated Globalization era since 1989, ultimately leading to the rise of new global multipolarity [21]. The last geopolitical concept was recognized surprisingly early among some of the leading think tank centres of the so called Collective [22] or Political West [23]. It appears to be a very distant event to the interdisciplinary health sciences at the first glance. Yet in reality, it is much more at the cross section of profound economic changes worldwide. Chinese contribution to the Global Medical Services and Device market, alongside with that of other leading BRICs nations, may be easily witnessed, with the Composite Annual Growth Rates of emerging Pharmaceutical Markets (CAGR) by far exceeding those of the established mature high-income economies worldwide [24]. Driven by return on investment principle, all major Big Pharma multinationals, for well over a decade, have grounded their long-term investment strategies oriented towards all of these Emerging economies [25]. To observe that scale of the evolution taking place it is enough to cite recent Brookings Institute report based on the International Monetary Fund data. It confirms that real GDP growth rates among the leading EM7 remained substantially higher before during and after the global economic recession 2007–2017 [26]. Professor Thomas Getzen, Founder of International Health Economics Association (IHEA) in his seminal paper with Mihajlo Jakovljevic, has confirmed that these shifts have already reflected heavily on world’s health expenditure landscape. LMIC countries participation in global health spending in terms of purchase power parity, has already grown in some indicators from almost 26.1% in 1995 to 39.7% in 2013, in only a span of 19 years [27].

These, and many other signs of huge changes in the global health sector and spending landscape worldwide, reveal the fact that CERA’s focus on LMICs and Emerging nations was indeed a prophetic vision a decade ago in a very different world of that time. What we may witness today on CERA development pathway? We may observe a consolidated and reputed journal attracting a sustainable number of decent quality and innovative submissions year to year around. Stringent external peer review criteria make its acceptance rates quite competitive among its open access counterparts. It holds an average Submission to First Decision so far at 116.3 days level. (average number of days between the date the manuscript was received and the first decision). The dynamics of CERA’s citations per document statistics continue to fluctuate around consolidated values since 2007, while the percentage of contributions from international collaborations represents the steady growing trend for well over a decade [28].

Yet among many strengths there are few setbacks such as the light contraction of the impact factor (from 1.788 in 2017 to 1.676 in 2018) and relative decrease of the SCImago Journal Rank Indicator (SJR) bibliographic indicator (from 0.888 in 2017 to 0.467 in 2018) [29]. What could be done to improve Journal’s efficiency and reputation as a platform for discussion among devoted health economists and policy makers in a global health care arena? Few assumptions might be considered as a success strategy for the next decade. Notably we must observe the competitive landscape from other major Publishers and Journals in HEOR interdisciplinary sciences [30]. This refers to the traditional academic journals and open access ones alike. Taking the time horizon of the past decade and dynamics of long-term trends of scientometric indicators, SCImago [31] and Clarivate Analytics indexing registries allow us insight into these underlying patterns [32]. We witness that most of traditional health economics journals have consolidated around their current impact factor values [33]. Few of the journals launched in late 1990s early 2000s with long-awaited impact factors (IF) obtained these in the medium range for common IF span in HEOR disciplines. Both categories exhibited a light tendency to decrease their IF in recent years. This has happened probably less due to the inner quality and innovativeness issues of these journals themselves [34], but far more due to a growing competition in global academic publishing arena primarily driven by Open Access business models [35]. The exception from this rule in terms of continued IF growth are a very few journals, particularly those with excellent bridges towards multinational pharmaceuticals and medical devices manufacturing industries worldwide [36].

Again, although important for CERA’s prestige, impact and citation rates have also a tendency towards a statistical bias when trying to explore the true outreach to the global scientific community. These limitations where emphasized in a large number of bibliographic sources over the recent decade. Furthermore, new scientometric indicators, were proposed [37], and some are undergoing broad adoption by academic community and publishers alike [38]. Typically, these are Source Normalized Impact per Paper (SNIP equals 0.636 for CERA in 2020) and SCImago Journal Rank (SJR equals 0.467 value in 2020) [39].

A decade ago CERA in a milestone Editorial article published in its 7th year our Journal confronted a major crossroad and a challenge. It was to expand its target audience far beyond dominantly Western OECD academic and industry based HEOR research centres towards the LMICs nations and the Emerging economies. Over the last decade, this aim was largely successfully achieved. Given the BMC policies on removing financial barriers against publishing for the corresponding authors based in low and lower middle-income countries, we managed outreach to many of these regions. CERA indeed became recognized as an established outlet for publishing high quality research on health financing and spending bottle neck inefficiencies facing Africa, Latin America and Southern Asia [40].

Yet today new challenges emerge. Many of them were recognized in the UN and WHO Health Financing reports and Global Burden of Disease health economics studies [41–43]. These mean that globally Donor Aid is surprisingly decreasing with a concerning consequences for provision of medical care for the most vulnerable world populations. This contraction taking place in recent years, is also worsened by the fact that most of this Aid is unevenly distributed in a way which heavily mismatches the true needs of the developing world [44]. Moreover, 90 + % is devoted exclusively to communicable diseases such as tuberculosis (TB), HIV/AIDS and malaria [45] with only a few percent to the noncommunicable diseases (NCDs) [46]. NCDs are indeed new pandemics of our Era and LMIC nations face the double burden of a non-liquidated pool of infectious diseases coupled with the high tide of diabetes, cancer, COPD, cardiovascular and mental health conditions. CERA’s further growth in the 2020s should exactly match these key health policy needs. So, we plan to establish ourselves as a forum for discussion on health financing sustainability faced with NCDs, global population ageing and technological innovation in medicine. This is even more pressing as pricing of pharmaceuticals continue to rise in a time of resource constraints and initiatives and calls for fair pricing mechanisms are clearly needed [47]. For this purpose, we remain open to a diversity of methodological frameworks in which such research hypothesis are answered. We welcome submissions ranging from traditional health economics evaluations such as cost-effectiveness/utility studies, budget-impact analysis but as well less widely adopted but relevant cost-consequence analysis and picturesque reviews in HEOR literature. We believe that in the third decade of XXI century the interdisciplinary science of health economics, will rejuvenate and come back to us younger than ever from its early historical roots almost a century ago. The spreading of economic globalization in several distinctive ways, either led by multinational business corporations or newly emerged Asian leadership, or both, is likely to make the challenges for contemporary health systems even far more serious. Furthermore the fourth industrial revolution of cyber physical systems, artificial intelligence technology and accelerated innovation in the field of E-Health, will probably change the workflow in medical care and inevitably transform the labour market in the upcoming decades. So, let us be up to the task. Let us provide academic centres, industry-sponsored drug and medicinal device development centres and governing authorities alike, with a powerful forum for debate on cost-effective resource allocation in the years to come.

## Data Availability

Not applicable.
